# Balancing Proliferation with Igκ Recombination during B-lymphopoiesis

**DOI:** 10.3389/fimmu.2014.00139

**Published:** 2014-04-02

**Authors:** Keith M. Hamel, Malay Mandal, Sophiya Karki, Marcus R. Clark

**Affiliations:** ^1^Department of Medicine, Section of Rheumatology, Gwen Knapp Center for Lupus and Immunology Research, The University of Chicago, Chicago, IL, USA

**Keywords:** B cells, lymphopoiesis, recombination, proliferation, epigenetics

## Abstract

The essential events of B-cell development are the stochastic and sequential rearrangement of immunoglobulin heavy (Igμ) and then light chain (Igκ followed by Igλ) loci. The counterpoint to recombination is proliferation, which both maintains populations of pro-B cells undergoing Igμ recombination and expands the pool of pre-B cells expressing the Igμ protein available for subsequent Igκ recombination. Proliferation and recombination must be segregated into distinct and mutually exclusive developmental stages. Failure to do so risks aberrant gene translocation and leukemic transformation. Recent studies have demonstrated that proliferation and recombination are each affected by different and antagonistic receptors. The IL-7 receptor drives proliferation while the pre-B-cell antigen receptor, which contains Igμ and surrogate light chain, enhances Igκ accessibility and recombination. Remarkably, the principal downstream proliferative effectors of the IL-7R, STAT5 and cyclin D3, directly repress Igκ accessibility through very divergent yet complementary mechanisms. Conversely, the pre-B-cell receptor represses cyclin D3 leading to cell cycle exit and enhanced Igκ accessibility. These studies reveal how cell fate decisions can be directed and reinforced at each developmental transition by single receptors. Furthermore, they identify novel mechanisms of Igκ repression that have implications for gene regulation in general.

## Introduction

Development of a diverse repertoire of peripheral B cells is dependent on the appropriate and ordered progression of B-lymphopoiesis. This process occurs through discrete developmental stages driven by the sequential rearrangement and expression of genes encoding the immunoglobulin heavy (Igμ) and then light chains (Igκ or Igλ). Successful expression of a functional Igμ capable of pairing with surrogate light chain (SLC) components and Igα/Igβ to form the pre-B-cell receptor (pre-BCR) at the cell surface is associated with a proliferative burst that expands the pool of pre-B cells expressing Igμ prior to cell cycle exit and the rearrangement of Igκ. Proliferation and recombination must remain mutually exclusive to maintain genomic integrity and prevent excessive cell death or oncogenesis through aberrant translocations. Recent work has begun to uncover the molecular mechanisms dictating these developmental stages. Of particular interest, is the integration and opposition of the IL-7R and pre-BCR signaling pathways along with the effect of downstream epigenetic modifications on Igκ loci rearrangement and early B-cell proliferation.

## B-Cell Development

Interactions with bone marrow (BM) stromal cells induce the differentiation of common lymphoid progenitor cells (CLPs), capable of generating B and T cells, into multipotential precursor–progenitor (pre–pro) B cells (1, 2). At this stage, initial Igμ rearrangements occur at diversity (D_H_) and joining (J_H_) gene segments (3). Pre–pro-B cells are not committed to the B-cell lineage as some developing T cells bear Igμ D_H_J_H_ rearrangements. Within IL-7 rich niches of the BM, pre–pro-B cells commit to the B-cell lineage through differentiation into progenitor (pro)-B cells expressing CD19 (4–6). IL-7 provides critical proliferative and survival signals needed to maintain the pool of pro-B cells. The hallmark event of pro-B cells is the completion of Igμ rearrangement with the addition of a variable (V_H_) region to the D_H_J_H_ segment. This process of recombination is mediated by the semi-random induction of double-stranded DNA breaks by the recombinase activating gene (Rag)-1 and Rag-2 proteins at recombination signal sequences (RSS) followed by non-homologous end joining (NHEJ) (7). Rag-mediated recombination of the antigen receptor loci is an essential and defining feature of B- and T-lymphopoiesis. Genetic mutation of the Rag genes results in severe combined immunodeficiency (SCID) in humans and mice (8–10).

Progression to the pre-B-cell stage of development is marked by the expression of a functional Igμ, due to in-frame rearrangement at one locus, which can pair with SLC components, VpreB and λ5, to form the pre-BCR at the cell surface (11). Early events following the expression of the pre-BCR serve to expand in number B-cell populations that have successfully rearranged Igμ (12). Not all Igμ chains effectively pair with SLC and therefore the pre-BCR checkpoint shapes the repertoire of Igμ chains selected into the small pre-B-cell pool (13). In mice deficient in SLC, cells that escape by rearranging immunoglobulin light chain are preferentially autoreactive (14). Furthermore, conferring defined self-reactivity rescues SLC deficiency (15). However, it is not clear if this means that the pre-BCR censors autoreactivity or if autoreactivity, and ligation by self-antigen, is required to complement SLC deficiency.

Following poly-clonal expansion, late (small) pre-B cells migrate away from proliferation-inducing IL-7 rich niches of the BM, exit cell cycle, and begin to rearrange Igκ genes (6). Final pairing of translated Igμ and Igκ form the antigen-specific BCR on immature B cells which are then subjected to the mechanisms of tolerance that diminish autoreactivity in the naïve repertoire. Although the necessity of the IL-7R and pre-BCR for B-lymphopoiesis has long been appreciated, recent work has begun to detail the molecular mechanisms and downstream interplay of these pathways that drive B-cell development.

## IL-7R and Pro-B Cells Fate

Signaling through the IL-7R, which is a heterodimer of the IL-7Rα chain and the common γ chain, is essential for proliferation and survival of pro- and pre-B cells. *In vitro* culture assays demonstrated that pro-B cells and not pre–pro-B cells proliferate in response to IL-7 (4). Accordingly, IL-7Rα-deficient mice demonstrate a significant impairment in B-lymphopoiesis beginning at the pro-B-cell stage (16–18). However, IL-7-deficient mice display a less severe defect in pro-B-cell development suggesting the IL-7Rα chain may participate in an additional signaling complex that compensates for loss of IL-7-induced signaling (17). Nonetheless, although pairing of IL-7Rα with alternative complexes may provide some compensation to IL-7-induced signaling, it is clear that the downstream components of the IL-7R pathway determine the pro-B-cell fate.

Through pairing with Janus kinase (JAK) 3 and JAK1, the IL-7R, upon activation, recruits and activates signal transducer and activator of transcription (STAT) 5a and b (19). STAT5 is critical for the biological effects of the IL-7R. B-cell development in mice deficient in both STAT5a and b is blocked at the pro-B stage, similar to IL-7Rα-deficient mice (20). Accordingly, constitutive activation (CA) of STAT5 in mice mostly restores B-lymphopoiesis in the absence of IL-7R signaling, while in humans, CA-STAT5 gene mutations have been identified in patients with acute lymphoblastic leukemia (21–23). Activated STAT5 primarily drives proliferation by inducing expression of the gene encoding cyclin D3, *Ccnd3* (23, 24). Pairing of cyclin D family members with cyclin-dependent kinases 4 and 6 (CDK4/6) during G_1_ activates retinoblastoma protein (Rb) family members and E2f transcription factors to induce upregulation of cell cycle genes and suppress cell cycle inhibitors p27^Kip1^ and p21^Cip1^ (25). Although both cyclin D2 and D3 are expressed during B-cell development, only cyclin D3 can be found in complexes with CDK4/6 in pro-B cells (26). Moreover, a defect in early B-cell development is found only in *Ccnd3*^−/−^ mice, while *Ccnd2*^−/−^ mice display a later defect in peripheral B-cell proliferation (24, 27, 28). In addition to proliferative signals, STAT5 maintains survival of developing B cells through induction of several pro-survival genes including Mcl1, Bcl2, and Pim1 (22, 29, 30). Therefore, IL-7R-mediated activation of STAT5 represents a critical event in the expansion and stability of early B cells populations.

Pro-B cells are both proliferating and rearranging Igμ genes (4). Recent studies have provided some insights into how these incompatible processes are segregated to distinct populations within the pro-B-cell pool (31, 32). For example, it has been demonstrated that the core machineries of recombination and proliferation are antagonistic. The Rag proteins are expressed in G0/G1 and are degraded in dividing cells at the transition from G1 to S phase (33). Cyclin A/CDK2 complexes induce cell cycle entry and inhibit the accumulation of Rag-2, while several CDK inhibitors, including p21^Cip1^, p27^Kip1^, and p57^Kip2^ induce Rag-2 expression (34). This is because the cyclin A/CDK2 complex phosphorylates threonine 490 of Rag-2 targeting it for degradation by Skp2 (35). Mutation of threonine 490 results in persistence of Ig recombination in proliferating cells and increases the prevalence of chromosomal translocations and lymphoid malignancies (36). Impaired NHEJ accompanied with defective DNA-damage-induced apoptosis also increases the occurrence of leukemogenesis. Mice with combined deficiencies of the pro-apoptotic protein p53 with either XRCC4 or Ku80, both members of the NHEJ machinery, develop IgH–Myc translocations that promote pro-B leukemia (37, 38). Therefore, separation of proliferation and recombination is crucial to the avoidance of excessive B cells’ death or development of B-cell leukemia.

**Figure 1:**
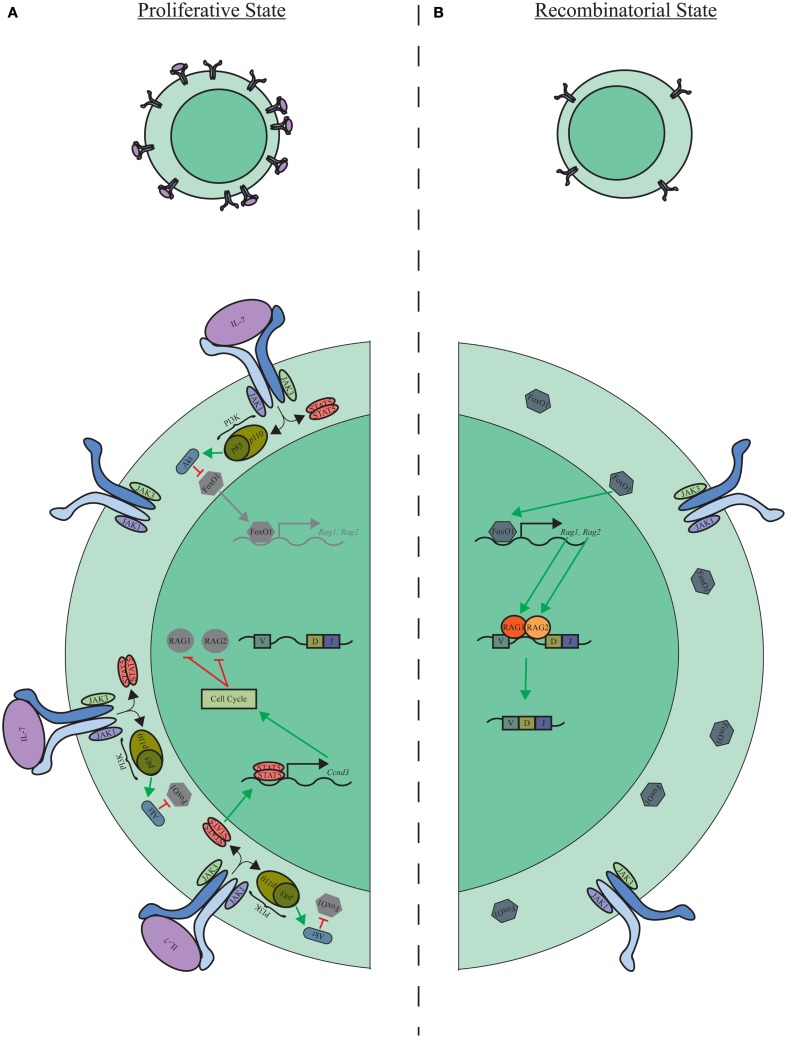
**Proliferative and recombinatorial states of pro-B cells**. **(A)** Elevated levels of IL-7R expression and signaling activate STAT5 and PI3K/Akt signaling modules, which enforce the proliferative program of pro-B cells while suppressing Igμ recombination. **(B)** Down modulation of the IL-7R is associated with a loss of proliferative signaling through STAT5 and PI3K/Akt and release of FoxO1, Rag-1, and Rag-2 suppression allowing progression of Igμ recombination.

It is also now clear that the pro-B-cell compartment is not homogeneous but contains subpopulations of cells that express relatively high or low levels of the IL-7R. Furthermore, in these populations, IL-7R expression levels correlate with intracellular-activated STAT5 (39). These findings suggest a dynamic model where pro-B cells shift from proliferation to recombination through the oscillation of IL-7R expression (Figure 1). In contrast to oscillating between IL-7R high and low states, it is also possible that pro-B cells sequentially progress through IL-7R high and low stages. The mechanism driving IL-7R downregulation in pro-B cells, however, is still unknown. One possibility is through asymmetric cell division, where the accumulation of IL-7R toward IL-7-producing stromal cells results in distal daughter cells inheriting less IL-7R on their surface, therein, providing a transient decrease in STAT5 activation and the initiation of V_H_–D_H_J_H_ rearrangement.

## Pre-BCR, Proliferation, and Igκ Rearrangement of Pre-B Cells

### Large pre-B cells

Cells transition to the pre-B-cell stage when Igμ pairs with SLC components, VpreB and λ5, along with the signaling module Igα/Igβ to form the pre-BCR at the cell surface. Initial expression of the pre-BCR is associated with a proliferative burst of early pre-B cells, also known as large pre-B cells, to expand the population of cells expressing a functional Igμ. Proper expression of the pre-BCR is critical to development as deficiencies of Igα, Igβ, or surface Igμ completely arrest B-lymphopoiesis while rearrangement and expression of Igκ inefficiently rescues SLC deficiency (40–43). Activation of the pre-BCR requires the non-immunoglobulin domain of λ5, which mediates aggregation of the receptor (44–46). Although receptor aggregation is required, it is not clear if receptor aggregation is an intrinsic property of λ5 or if the SLC enables recognition of one or more selecting ligands within the BM (44, 47). Putative selecting ligands identified within the BM including heparin sulfate and galectin-1 have been suggested as natural ligands (48–50).

Concurrent to pre-BCR expression, large pre-B cells maintain IL-7R expression. It is within large pre-B cells that an additional downstream target of IL-7R signaling important for B-cell development, the phosphoinositide 3-kinase (PI3K) pathway, plays a role (51, 52). The absence of PI3K has a definitive effect on peripheral B-cell proliferation, and selective deletion of the regulatory subunit p85α or the combined catalytic subunits p110α and p110δ result in impairment of B-lymphopoiesis (53–55). However, the effects of PI3K on early B-cell proliferation appear to be within the initial proliferative events of pre-B cells, not pro-B cells. Deficiencies in p85α or PTEN, a negative regulator of PI3K does not affect the number of pro-B cells in cycle, and the defect in development in p110α- and p110δ-deficient mice begins at the pre-B-cell stage (26, 52). Compared to cycling pro-B cells, large pre-B cells are indeed larger in size and display a heightened rate of proliferation (4). PI3K may be required in large pre-B cells to support increased protein synthesis and rapid cell division through increased glucose uptake and glycolytic activity by activated Akt, downstream of PI3K (56–58). Coincidently, Akt is capable of enhancing survival by inhibiting pro-apoptotic pathways through direct repression of BAD and also indirectly by suppressing FoxO transcription factors, which induce Bim (59–62).

The pre-BCR is expressed on large pre-B cells and therefore has been thought to enhance proliferation in response to IL-7R signaling. Among, the signaling pathways common to the BCR and the IL-7R in the periphery, PI3K was an attractive candidate for any synergy that might occur between the two receptors. However, the pre-BCR does not efficiently couple to PI3K. Transfection of *Rag-2*^−/−^ pro-B cells in the presence of IL-7 with a prearranged, functional Igμ resulting in pre-BCR expression does not increase phospho-Akt activation and phospho-Akt levels are similar in pro and large pre-B cells (52). Furthermore, deletions of the genes encoding several pre-BCR downstream signaling components, including BLNK (SLP-65), Btk, and phospholipase Cγ2 (PLCγ2), result in a developmental block at the cycling pre-B-cell stage (63–65). Finally, re-expression of BLNK in deficient cells induces cell cycle arrest and Igκ rearrangement (66). These observations indicate that the pre-BCR signals cell cycle exit rather than proliferation.

Therefore, the mechanisms driving the pre-B-cell proliferative burst remain unclear. It is possible that in pre-B cells, the pre-BCR has two signaling states, one pro-proliferative and one anti-proliferative (52, 67). However, the downstream effectors of such a pre-BCR-dependent proliferative pathway have yet to be identified. Alternatively, signaling mechanisms occurring independently of the pre-BCR could enhance IL-7R-mediated proliferation.

In addition to driving proliferation, signals through the IL-7R, and the downstream activation of STAT5, potently repress Igκ recombination (68). Activated STAT5 binds as a tetramer to a critical E-box-containing enhancer region of Igκ, the intronic enhancer (Eκ_i_), and tetrameric binding enables recruitment of the polycomb repressive complex (PRC2), which represses accessibility of the Igκ region (69). Additionally, PI3K–Akt activation by the IL-7R represses recombination through indirect downregulation of Rag proteins (52). FoxO transcription factors induce Rag-1 and Rag-2 expression, however, repression of FoxO by the PI3K–Akt module inhibits Rag protein expression and inhibits recombination (70, 71). Therefore, beyond the intrinsic regulation of Rag proteins by the cell cycle machinery as described above, in large pre-B cells, IL-7R signaling through STAT5, and the PI3K–Akt module, further enforce proliferation while suppressing pre-BCR-induced recombination.

### Small pre-B cells

**Figure 2:**
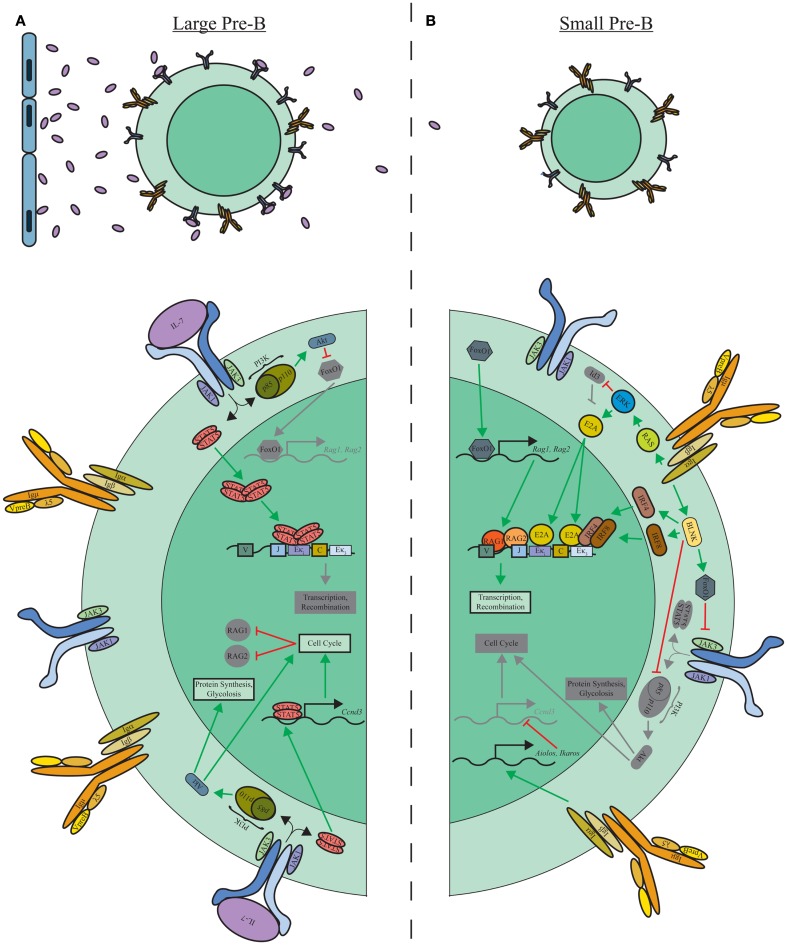
**IL-7R and pre-BCR mediated transition of large pre-B to small pre-B cells**. **(A)** Localization of large pre-B cells near IL-7-producing stromal cells maintains IL-7R-induced proliferation through STAT5 and PI3K/Akt signaling modules. Additionally, tetrameric STAT5 reinforces inhibition of Igκ recombination through direct binding to Eκi. **(B)** Migration away from IL-7-rich niches limits IL-7R signaling allowing pre-BCR-induced Ras/ERK and BLNK signaling modules to promote E2A and IRF4/IRF8 induction. Binding of these transcription factors to Igκ enhancer elements enables recombination in small pre-B cells. Additionally, the BLNK module, along with Aiolos and Ikaros, downstream of the pre-BCR inhibit proliferation by repressing IL-7R expression, PI3K/Akt activation, and *Ccnd3* transcription.

The transition from highly proliferative large pre-B cells to small resting pre-B cells undergoing Igκ recombination is a pivotal point in normal B-lymphopoiesis. This transition is controlled by the signaling cascades downstream of the IL-7R and pre-BCR (Figure 2). As described below, the pre-BCR orchestrates Igκ recombination, but cannot do so while the IL-7R is transmitting signals (23, 52, 68). First cells must escape IL-7 signaling, presumably through migration toward IL-7 low niches of the BM (6). Interestingly, upregulation of the interferon regulatory factor (IRF)-4 by the pre-BCR induces the expression of the chemokine receptor CXCR4 (68). The potential presence of the CXCR4 ligand, CXCL12, outside of IL-7 niches, may provide a mechanism by which early events of the pre-BCR enables movement into relatively IL-7-deficient niches and transition from proliferation-inducing signals (IL-7R) to those driving recombination (pre-BCR).

The opening of the Igκ locus by the pre-BCR is predominately accomplished through activation of the Ras/Erk pathway (23, 72). Activated Erk induces E2A and inhibits the E2A repressor Id3 leading to an accumulation of free E2A within the nucleus (23, 73) that then binds the Eκ_i_ and the Igκ 3′ enhancer (Eκ_3_) (23). Escape from IL-7 signaling relieves tetrameric STAT5 occupancy of Eκ_i_, allowing E2A to bind, which promotes accessibility of the Igκ loci for transcription and recombination (69). Genetic targeting of the E-boxes within Eκ_i_ has demonstrated the importance of E2A recruitment in Igκ recombination (74).

In addition to de-repressing Igκ, loss of IL-7R signaling enhances specific pre-BCR-dependent and -independent mechanisms important for Igκ recombination. Loss of IL-7R-induced PI3K–Akt activation results in increased FoxO expression. FoxO1 directly binds the Rag-1 and -2 genes and induces their expression (70). FoxO also binds and induces expression of the Syk and BLNK genes (52). The Syk/BLNK module induces the transcription factors IRF4 and 8, which bind the 3′ Igκ enhancer (Eκ_3_) and enhance Igκ accessibility (68, 75, 76). Furthermore, downstream of BLNK, activation of p38 MAP kinase further enhances FoxO activation thereby setting up a feed-forward loop that reinforces commitment to Igκ recombination (52).

Pre-B-cell receptor signals additionally repress the proliferative program. FoxO1 represses surface expression of IL-7R in pre-B cells, while BLNK inhibits PI3K/Akt activation (52, 71). Pre-BCR signals also induce the expression of the transcription factors Aiolos and Ikaros (77, 78). These factors impede cell cycle by repression of Myc and cyclin D3 gene expression (23, 78). Accordingly, conditional deletion of Ikaros at the pro-B-cell stage of development results in a severe block in B-lymphopoiesis with an accumulation of cycling large pre-B cells (79). Ikaros might have a direct role in Igκ recombination although the mechanisms remain to be defined (79). Collectively, downstream of the IL-7R and pre-BCR, these networks of feed-forward and feed-back mechanisms mediate the transition from proliferation to recombination and ensure sharp demarcation between each developmental state (80).

## Epigenetic Regulation of Igκ Accessibility and Recombination

### IL-7R and pre-BCR imposed regulation of Igκ accessibility

**Figure 3:**
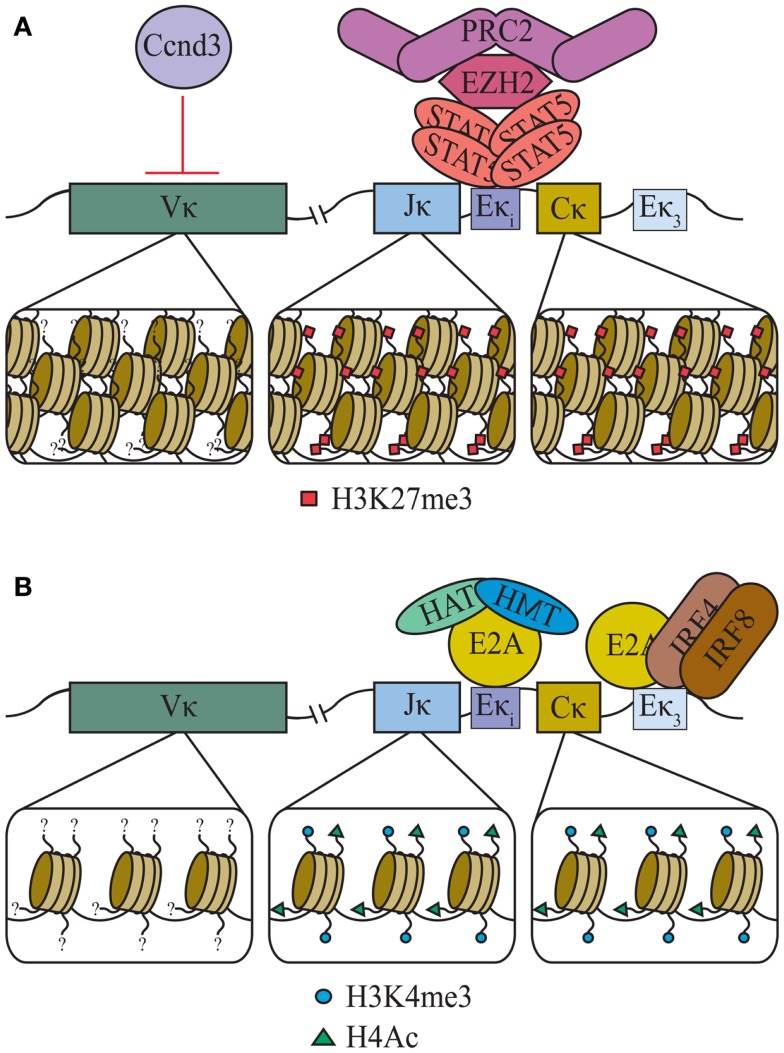
**Epigenetic regulation of the Igκ loci**. **(A)** In large pre-B cells, downstream of the IL-7R, tetrameric STAT5 directly binds at Eκ_i_ as a tetrameric complex. This both inhibits E2A binding and recruits the methyltransferase EZH2 and polycomb repressive complex 2 (PRC2) which decorates Jκ and Cκ with H3K27me3. Additionally, through an unknown mechanism, Cyclin D3 (Ccnd3) restricts Vκ segments’ accessibility. **(B)** Loss of IL-7R signaling in small pre-B cells leads to a loss of tetrameric STAT5 at Eκ_i_ which allows E2A binding and the recruitment of histone methyltransferases (HMT) and histone acetyltransferases (HAT). The resulting H3K4me3 and H4Ac marks open Jκ and Cκ to transcription and recombination.

Chromatin structure and accessibility are fundamental to B-cell development. Recent evidence indicates that, at least in part, accessibility of Ig genes is determined by post-translational epigenetic modifications of regional histone cores. Accessibility to recombination correlates with transcription (81) and indeed the primary effectors of epigenetic remodeling are transcription factors. It has become apparent that both STAT5 and E2A regulate Igκ accessibility by determining the epigenetic landscape of the locus in pre-B cells (Figure 3). Initially, tetrameric STAT5, downstream of the IL-7R, recruits the histone methyltransferase Ezh2, which decorates the Igκ locus with repressive histone 3 lysine 27 trimethylation (H3K27me3) marks (69). Following release from STAT5-mediated repression of Igκ, E2A can access Eκi, and marks the flanking Jκ and Cκ segments with activating H3K4 trimethylation (H3K4me3) and H4 acetylation (H4Ac) to promote an open chromatin structure (69, 82).

Interestingly, the above mechanisms of epigenetic regulation apply only to Jκ and Cκ and do not extend to the extensive Vκ regions (69). In fact, the Vκ regions are relatively devoid of any measured post-translational histone modifications identified for Cκ and Jκ [unpublished data and (83)]. Surprisingly, Vκ transcription is repressed by cyclin D3, through mechanisms that do not involve direct DNA binding (26). Instead, it appears that nuclear matrix-associated cyclin D3, and not that fraction associated with CDK4/6, represses Vκ. The mechanisms by which cyclin D3 regulates Vκ transcription are not known, but might include controlling access to RNA polymerase II or nuclear positioning (84, 85). Regardless of mechanism, repression of Vκ accessibility by cyclin D3 provides a direct link between cell cycle transit and repression of Igκ recombination.

### Rag-mediated recombination depends upon epigenetic modifications

Recombination events at Igκ are also dependent on an open chromatin structure for accessibility of Rag proteins to RSS sites. RAG-mediated cleavage at RSS sites is restricted by a closed nucleosome structure (86–88). Histone modifications associated with open chromatin structures, including H3K4me3, histone 3 lysine 36 trimethylation (H3K36me3), H3Ac, and H4Ac correlate with recombination (89–91). Additionally, the recruitment of Rag-2 is dependent on the Rag-2 PHD domain binding to H3K4me3 (92, 93). The epigenetic regulation of JκCκ, and the recruitment of RAG-2 to the marks of open chromatin, is consistent with current concepts that the JκCκ region serves as the site of recombination (94). Furthermore, the JκCκ region is anchored to the nuclear matrix and anchoring is necessary for efficient Igκ recombination (95). This suggests that the recombination platform is relatively fixed and Vκ segments are recruited to it.

Although histone modifications at Jκ and Cκ have been associated with recombination and Rag-2 recruitment *in vivo*, there is no direct evidence that these modifications alone are capable of inducing RSS accessibility. In fact, *in vitro* experiments have demonstrated that hyperacetylation of histones is unable to overcome nucleosome-induced restriction of RSS sites and allow Rag-mediated recombination (87, 96). However, these extracellular *in vitro* experiments may lack additional lineage or stage-specific factors needed to translate epigenetic modifications into open chromatin. One such factor might be the SWI/SNF complex which can read specific epigenetic marks and open immunoglobulin gene loci for recombination (83, 97).

## Concluding Remarks

Recent observations have revealed that the IL-7R and the pre-BCR regulate complex networks of signaling and transcription cascades that direct and reinforce either pre-B-cell proliferation or Igκ recombination. Central to understanding these networks is the clear demonstration that the IL-7R induces proliferation and represses Igκ recombination and these biological activities are diametrically opposed by the pre-BCR. However, several questions still remain. For instance, if IL-7R signaling is constant in pro- and pre-B cells, and the pre-BCR does not provide a proliferative signal, what then is driving the large pre-B-cell proliferative burst? Additionally, although much effort has begun to describe how fate-determining transcription factors and epigenetic modifiers prime the required epigenetic landscape, little is known about the “readers” of these marks that impose and specify B-cell developmental events. The precise relationships between Igκ transcription and recombination are unclear. Moreover, in the absence of epigenetic modifications, how is Vκ accessibility regulated? Further research into the molecular mechanisms that target and regulate the recombinatorial machinery to specific sites of the Ig loci will be critical for understanding normal and pathogenic B-lymphopoiesis.

## Conflict of Interest Statement

The authors declare that the research was conducted in the absence of any commercial or financial relationships that could be construed as a potential conflict of interest.
